# Synthesis and Characterization of Solvent-Complexes of *per*-Hydroxy Pillar[5]arene and Pillar[5]quinone: Experimental and Computational Insights

**DOI:** 10.1021/acsomega.5c10756

**Published:** 2026-03-04

**Authors:** Venkatesh Bollabathini, Quoc D. Ho, Eva Rauls, Kåre B. Jørgensen

**Affiliations:** † Department of Chemistry, Bioscience and Environmental Engineering, Faculty of Science and Technology, 56627 University of Stavanger, P.O. Box 8600 Forus, N-4036 Stavanger, Norway; ‡ Department of Mathematics and Physics, Faculty of Science and Technology, University of Stavanger, P.O. Box 8600 Forus, N-4036 Stavanger, Norway

## Abstract

When the macrocycles *per-*hydroxy pillar[5]­arene (P[5]­A–OH) and pillar[5]­quinone (P[5]­Q) are recrystallized from suitable solvents like acetone and 1,1,2,2-tetrachloroethane (TCE), respectively, the solvent molecules form host–guest complexes in a 1:2 stoichiometric ratio. These complexes survive prolonged exposure to vacuum at room temperature. Herein, we report improved yields for the preparation of 1,4-dimethoxypillar[5]­arene (DMP[5]­A) and its derivatives P[5]­A–OH and P[5]­Q, including detailed recrystallization procedures. The complexation behavior of these solvent molecules was investigated by ^1^H NMR and Infrared (IR) spectroscopy, interpreted with *ab initio* calculations. The *ab initio* calculations demonstrate that P[5]­A–OH encapsulates two acetone molecules within its cavity, whereas P[5]­Q binds with two TCE molecules outside the cavity. These complexes are stabilized by a combination of hydrogen bonding and CH−π interactions. IR spectroscopy confirmed the absorption of acetone by P[5]­A–OH and displayed a blue-shifted C=O stretch (1779 vs 1727 cm^–1^ in free acetone) and red-shifted O–H modes (3384/3622 cm^–1^). Our charge transfer and structural analysis indicate that the C=O bond of acetone is contracted (1.24 Å→1.22 Å) due to enhanced polarization. For P[5]­Q, TCE absorption is driven by hydrogen bonds, evidenced by the shift of C–H IR peaks near 3000 cm^–1^ and red-shifted C–Cl/C=O vibrations. Our study highlights the critical role of functional groups and noncovalent interactions in guest orientation, providing key insights for designing supramolecular materials.

## Graphical abstract

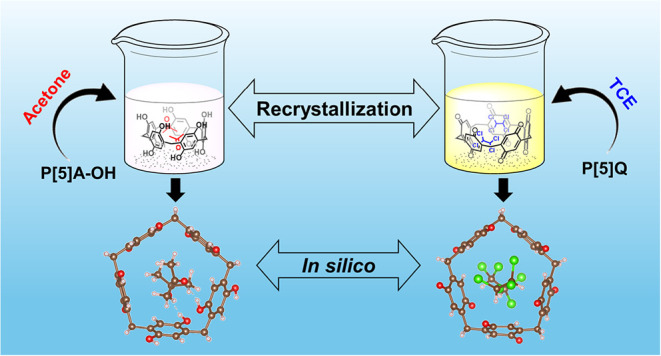

## 1 Introduction

Macrocyclic compounds are widely recognized as essential molecular receptors due to their structurally preorganized cavities and multiple noncovalent binding sites that facilitate host–guest complexation.^1^ Extensively investigated macrocyclic hosts like crown ethers, cyclodextrins, and calixarenes have demonstrated their ability to encapsulate a diverse range of guest molecules.^2^ Since the seminal contribution by Ogoshi et al. in 2008,^3^ pillar­[*n*]­arenes (P­[*n*]­As) have rapidly gained interest as key synthetic macrocyclic platforms to explore the host–guest chemistry.^4^ As a relatively recent addition to the macrocyclic host family, P­[*n*]As possess highly symmetrical structures with electron-rich cavities and versatile functionality. These characteristics make them well-suited as molecular hosts for the formation of supramolecular host–guest inclusion complexes in solution.^5^ Pillar[5]­quinone (P[5]­Q),^6^ the earliest known quinone-based analogue of pillar[5]­arene (P[5]­A), reflect P[5]­A’s characteristic *C*_5_-symmetry and offers a unique blend of structural rigidity and redox functionality.^7^ Notably, P­[*n*]­Qs are composed of electron-deficient *p-*benzoquinone units, differentiating them from P­[*n*]­As, whose electron-rich cavities originate from their aromatic phenylene units. The electronic reversal behavior of P­[*n*]­Qs would enhance their binding affinity toward guest molecules bearing electron-rich halogen atoms.^8^ Although P[5]­Q features a distinctive electronic framework, its potential in host–guest chemistry has not been extensively explored.

Host–guest inclusion complexes inevitably play a pivotal role in the chemical behavior of P[5]­A derivatives. In general, the inclusion complexes are formed through the interaction of host and guest molecules, where the host cavity promotes the guest inclusion via multiple noncovalent driving forces, including hydrogen bonding, van der Waals forces, electrostatic, and hydrophobic interactions.^9–12^ In addition, the inner cavity size of the host molecule is a critical structural feature that influences the selectivity of its host–guest binding behavior. The combination of experimental studies and computational modeling will enable the determination of well-defined conformations of the synthetic host–guest complexes, providing critical insights into their structural features.

Previous literature reports have already shown that upon recrystallization of *per-*hydroxy pillar[5]­arene (P[5]­A–OH) in acetone^13^ and P[5]­Q in TCE,^14^ the corresponding solvent molecules formed complexes with the host compounds (Figure 1). In the present study, we report the synthesis of macrocyclic precursor 1,4-dimethoxypilar[5]­arene (DMP[5]­A) and its subsequent derivatives P[5]­A–OH and P[5]­Q with detailed recrystallization procedures. These complexes were studied by ^1^H NMR and IR spectroscopy, followed by verification of their structural features by computational simulations for further validation.

**Figure 1:**
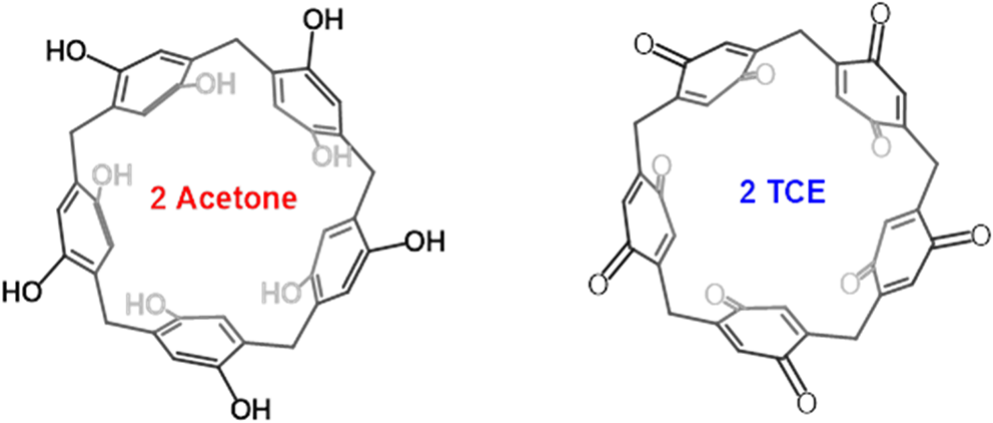
Complexes of P[5]­A–OH and P[5]­Q with solvent molecules, acetone, and TCE, respectively.

Density Functional Theory (DFT) has proven to be a powerful tool for investigating material properties, contributing to enhanced applications in areas such as optoelectronic devices, gas sensing, solar cells, photo catalysis, and gas adsorption.^15–21^ Tan and co-workers^22,23^ were among the first to investigate CO_2_ adsorption on pillar[5]­arene-based supramolecular organic frameworks (SOFs). Their research showed that these materials can adsorb CO_2_ well and are highly selective in separating the CO_2_ from different gas mixtures. Similarly, Wang et al.^24^ studied SOFs of cocrystallized pillararenes and 4,4′-bipyridine, demonstrating notable selectivity for CO_2_ over N_2_. Indeed, previous simulation studies^25–28^ using *ab initio* calculations have highlighted the significant potential of P[5]As for CO_2_ capture, examining in detail how factors such as cavity size and functional group modifications influence adsorption performance.

## 2 Methods

### 2.1 Experimental Section

The precursor DMP[5]­A^13^ and its derivatives P[5]­A–OH and P[5]­Q^29^ were synthesized according to the previously reported or modified procedures. For further purification, DMP[5]­A was recrystallized from a 1:1 solvent mixture of chloroform and acetone, whereas P[5]­A–OH and P[5]­Q were recrystallized from acetone and 1,1,2,2-tetrachloroethane (TCE), respectively (detailed procedures are provided in the Supporting Information). The compounds were subjected to a high vacuum overnight to remove any weakly associated solvent molecules. The complexation behavior of acetone and TCE with recrystallized P[5]­A–OH and P[5]­Q was studied by ^1^H NMR (400 MHz Bruker Advance III spectrometer) and IR spectroscopy (Attenuated Total Reflectance (ATR) on an Agilent Carey 630 FTIR).

### 2.2 Computational

Initial structural relaxations were performed using the SCC-DFTB method (DFTB+ 22.1)^30–33^ with the *mio-1–1* parameter set,^32^ suitable for C, O, and H atoms for the calculations of P[5]­A–OH and acetone. In the case of P[5]­Q with TCE, the *3ob-3–1* parameter set^34^ was used for C, O, H, and Cl. Valence orbitals included 2s and 2p (C, O, and Cl) and 1s (H), with core electrons treated as frozen. Long-range van der Waals interactions were accounted for using the SimpleDftD3 scheme, benchmarked against DFT-D3 with Becke-Johnson damping.^27,35,36^ This approach offers computational efficiency and avoids BSSE, while maintaining DFT-level accuracy.

Phonon calculations were performed using the finite displacement method implemented in the Vienna Ab initio Simulation Package (VASP 5.4.4)^37–39^ in combination with the PHONOPY code.^40,41^ Atomic displacements of 0.01 Å were applied to construct the dynamical matrix. IR spectra were derived from the Γ-point vibrational modes. Bader charge analysis^42^ was also computed using VASP and VTST tools. The calculations employed the projector augmented-wave (PAW) method with a plane-wave energy cutoff of 400 eV, the Perdew–Burke–Ernzerhof (PBE)^43^ functional for exchange-correlation, and DFT-D3 corrections to account for van der Waals interactions, ensuring accurate representation of both electronic and vibrational properties.

## 3 Results and Discussion

The precursor, DMP[5]­A was synthesized from its monomer, 1,4-dimethoxybenzene (DMB), using a well-established Lewis-acid catalyzed cyclization approach^13^ in excellent yields (up to 94%) (Scheme 1). In the next step, the *O*-demethylation of DMP[5]­A was carried out with boron tribromide (BBr_3_) to afford the corresponding hydroxylated derivative, P[5]­A–OH.^44^ P[5]­Q was synthesized in high yields through a direct oxidative transformation of DMP[5]­A using ceric ammonium nitrate (CAN).^29^

**Scheme 1:**
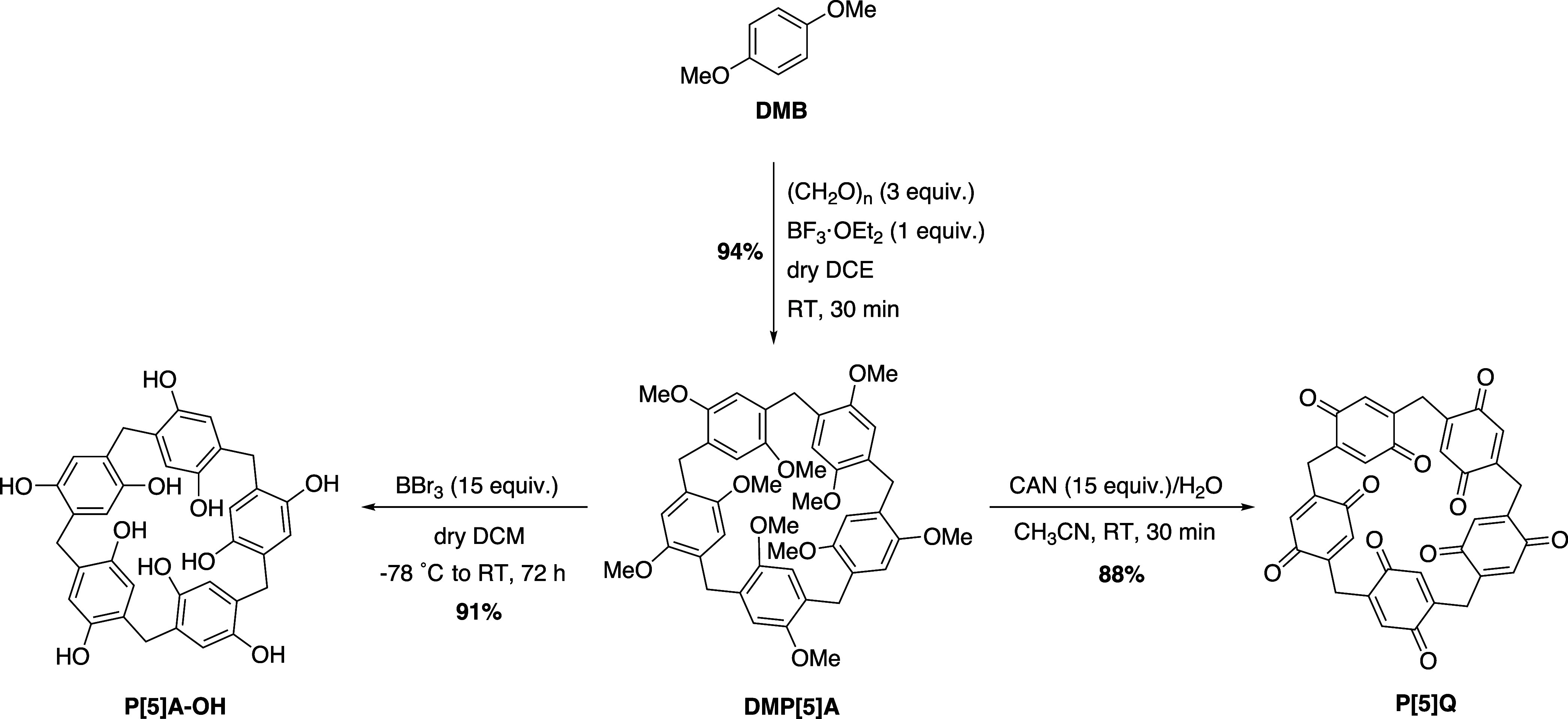
Synthesis of P[5]­A–OH and P[5]­Q from DMP[5]­A

### 3.1 ^1^H NMR Study

Following the synthesis and recrystallization-based purification of P[5]­A–OH and P[5]­Q, structural characterizations were performed by ^1^H NMR spectroscopy in DMSO-*d*_6_ and TFA-*d*, respectively. The assigned shift values for both compounds are summarized in Table 1. ^1^H NMR spectroscopy provides a superior approach over other nonseparative analytical techniques for investigating host–guest complexes, owing to its ability to simultaneously observe both host and guest molecules at the atomic level.^4,45^ Therefore, ^1^H NMR has been widely adopted for studying host–guest complexes formed by P­[*n*]As and their derivatives.

**Table 1:** Observed ^1^H Chemical Shift Values of Crude and Recrystallized Forms of P[5]­A–OH and P[5]­Q Compounds

	chemical shift (δ, ppm)			
position	P[5]A–OH (crude) (DMSO-*d*_6_)	P[5]A–OH (recrystallized) (DMSO-*d*_6_)	P[5]Q (crude) (TFA-*d*)	P[5]Q (recrystallized) (TFA-*d*)
H_Bridge_	3.43	3.43	3.71	3.70
H_Aromatic_	6.58	6.57	7.03	7.02
H_Hydroxy_	8.42	8.44		

The ^1^H NMR spectrum of P[5]­A–OH (recrystallized) exhibits its characteristic peaks at 3.43 (10 H), 6.57 (10 H), and 8.44 (10 H) ppm, corresponding to the methylene bridge, aromatic, and hydroxy protons, respectively (Figure 2b). The chemical shifts of neither the ^1^H nor ^13^C spectra (Supporting Information) were affected by the recrystallization process. Compared to the ^1^H NMR spectrum of P[5]­A–OH (crude) (given in the Supporting Information), a new peak from acetone was observed at 2.08 ppm. Careful integration of this peak revealed 12.01 protons, corresponding to exactly 2 molecules of acetone per pillararene molecule, which were incorporated into the compound during recrystallization. The similar observation has been made by single crystal X-ray analysis as reported in the previous literature.^13^ These results suggest that two acetone molecules were closely associated with P[5]­A–OH via noncovalent interactions, likely within the cavity.

**Figure 2:**
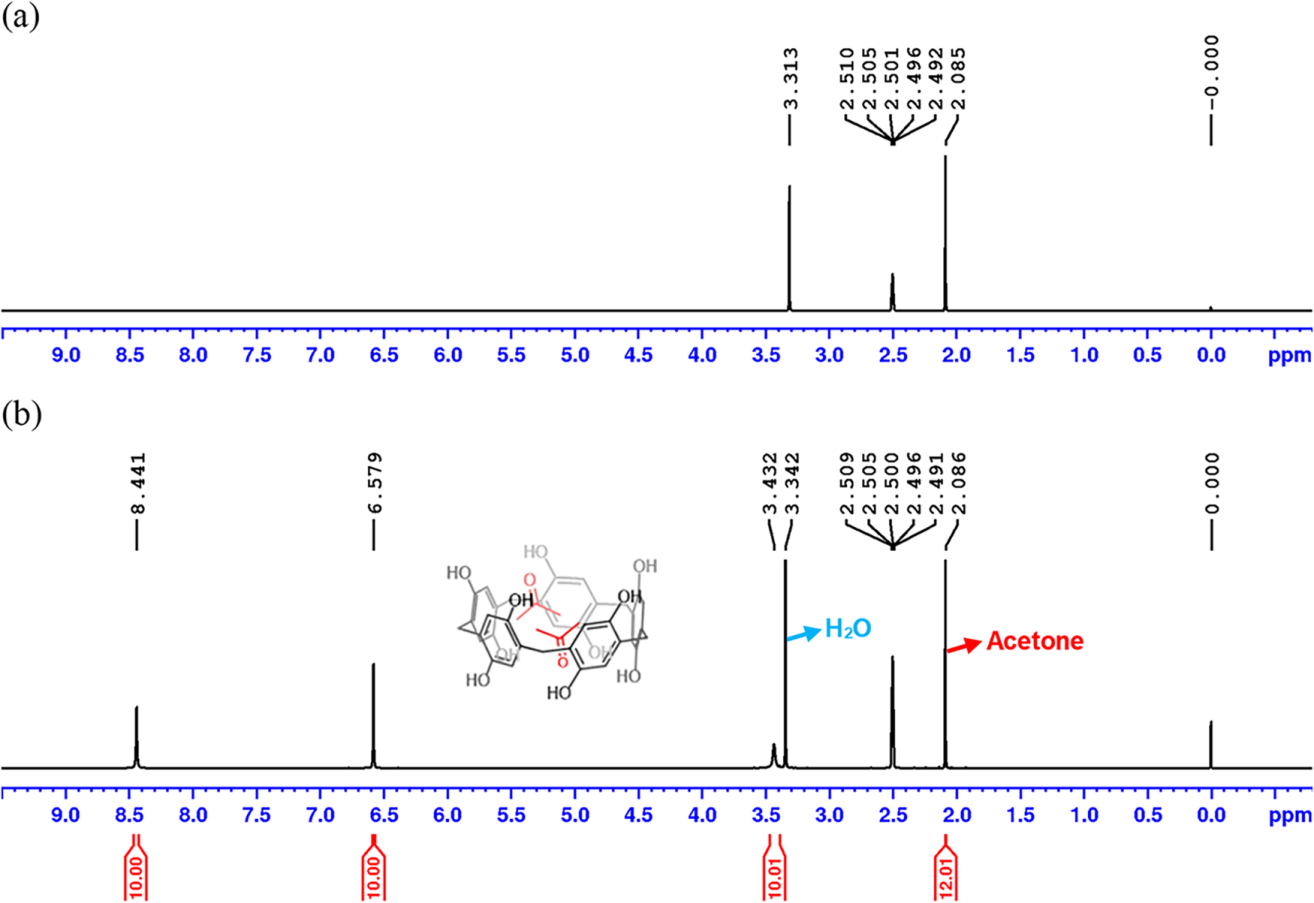
^1^H NMR spectra of (a) acetone alone and (b) P[5]­A–OH (recrystallized) in DMSO-*d*_6_ (solvent peak at 2.50 ppm).

A control spectrum of acetone alone in DMSO-*d*_6_ (Figure 2a) gave the same shift value of 2.08 ppm as that observed in the pillararene sample. Encapsulation of guest molecules within the pillararene supramolecular systems typically alters the guest’s electronic environment significantly, resulting in large chemical shift variations relative to its free state.^46–49^ However, pillararene host–guest complexes are known to have large solvent effects where the binding constants are reduced in polar solvents.^50^ The lack of changes in the chemical shifts indicates that guest-specific interactions are negligible and those acetone molecules undergo dynamic exchange in DMSO. Due to the inadequate solubility of P[5]­A–OH in nonpolar solvents, a polar solvent had to be employed for its NMR analysis.

The ^1^H NMR spectrum of P[5]­Q (recrystallized) revealed the characteristic peaks at 7.02 (10 H) and 3.70 (10 H) ppm, attributed to the protons of benzoquinone and the methylene bridge within the macrocyclic framework (Figure 3b). In addition, careful integration of this spectrum revealed a peak from TCE at 5.92 ppm with an integration area corresponding to four protons, indicating the presence of 2 molecules of TCE per pillarquinone molecule. Based on the observed spectral data, the interaction between TCE and P[5]­Q is governed by noncovalent forces, likely occurring at the macrocyclic cavity.

**Figure 3:**
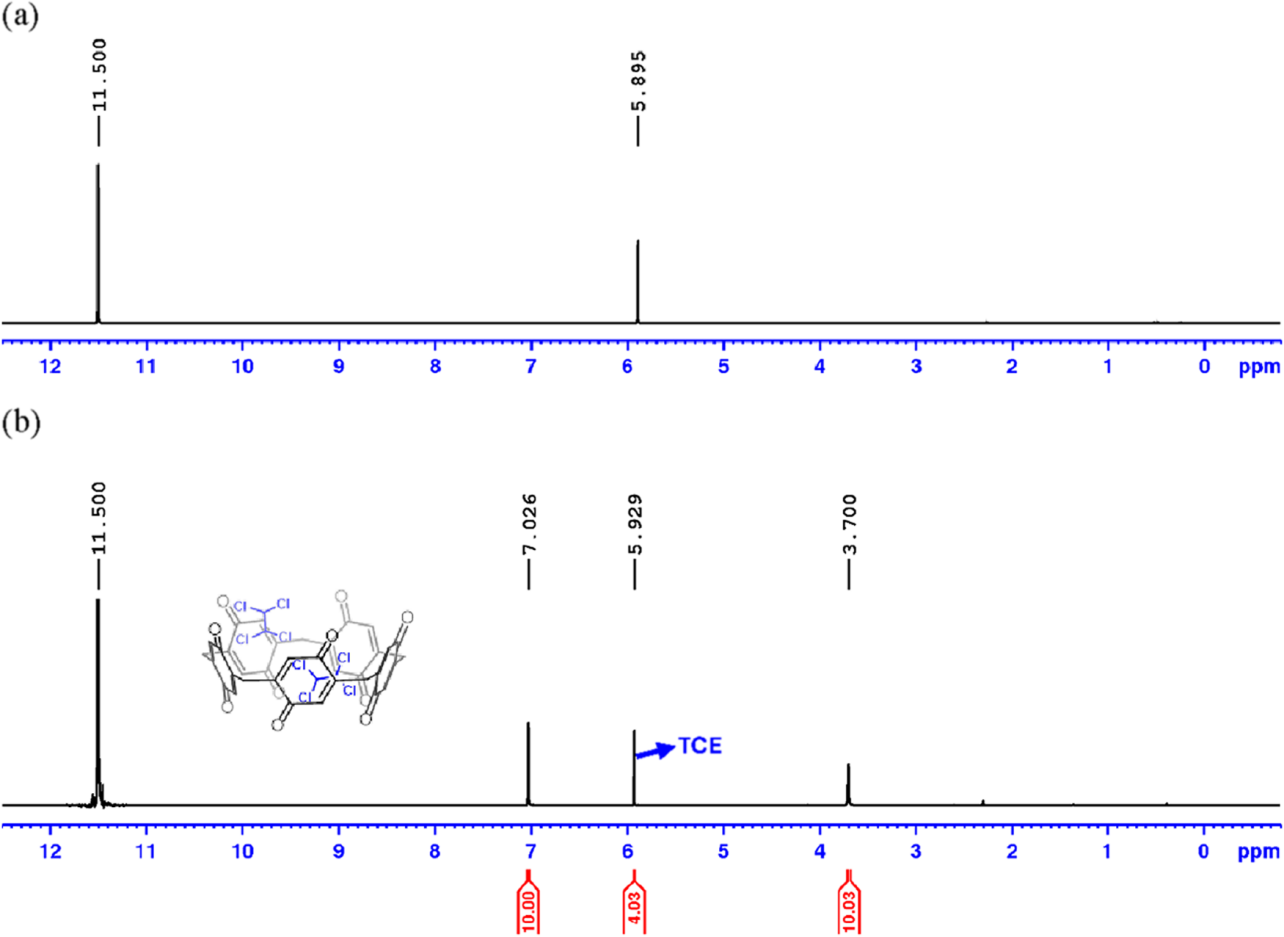
^1^H NMR spectra of (a) TCE alone and (b) P[5]­Q (recrystallized) in TFA-*d* (solvent peak at 11.50 ppm).

A control ^1^H NMR spectrum of TCE alone in TFA-*d* (Figure 3a) was performed and compared with that of P[5]­Q (recrystallized). Interestingly, the ^1^H NMR spectrum displays a small but significant chemical shift difference between TCE alone at 5.89 ppm and TCE bound to P[5]­Q (recrystallized) at 5.93 ppm. These observations suggest that the guest molecules exhibit some affinity with the P[5]­Q cavity, which slightly hinders the dynamic exchange in TFA.

### 3.2 Infrared Spectroscopy of P[5]­A–OH

IR measurements were conducted on the solids in an ATR cell to investigate the formation of host–guest complexes. It serves as an important analytical tool that provides additional insight by detecting characteristic vibrational changes of complexes associated with host–guest interactions.

For investigation of the P[5]­A–OH electronic and structural changes after acetone absorption, experimental and simulated IR spectra were analyzed and plotted (Figure 4). Vibrational modes in the 1180–1600 cm^–1^ region were assigned to C–C stretching vibrations. The acetone peak is measured at 1691 cm^–1^, which is related to the stretching of the acetone C=O bond. DFT simulated IR spectra (black) predict the C=O peak at 1779 cm^–1^. In the region of 3000 cm^–1^, 2 peaks were observed: a lower-energy band at 2934 cm^–1^ and a higher-energy band at 3073 cm^–1^. The IR spectrum simulated with DFT found a group of peaks in this region with the two strongest peaks at 2939 and 3039 cm^–1^. They are assigned to C–H stretching with the lower frequency belonging to the C–H stretch in the methylene bridge, while the other in higher energy corresponds to the aromatic C–H stretching in benzene rings. In the higher frequency region of the IR spectrum (blue), the experimental data reveals a very broad peak at 3240 cm^–1^, which are attributed to the stretching vibrations of hydroxy (−OH) groups. These relatively lower frequencies suggest the presence of strong hydrogen bonding, which typically causes a red shift (lowering) in the O–H stretching frequencies due to interactions with surrounding molecules or the crystal environment. The *ab initio* calculations predict O–H stretching peaks at 3383 and 3622 cm^–1^, both of which are higher than the experimental values.

**Figure 4:**
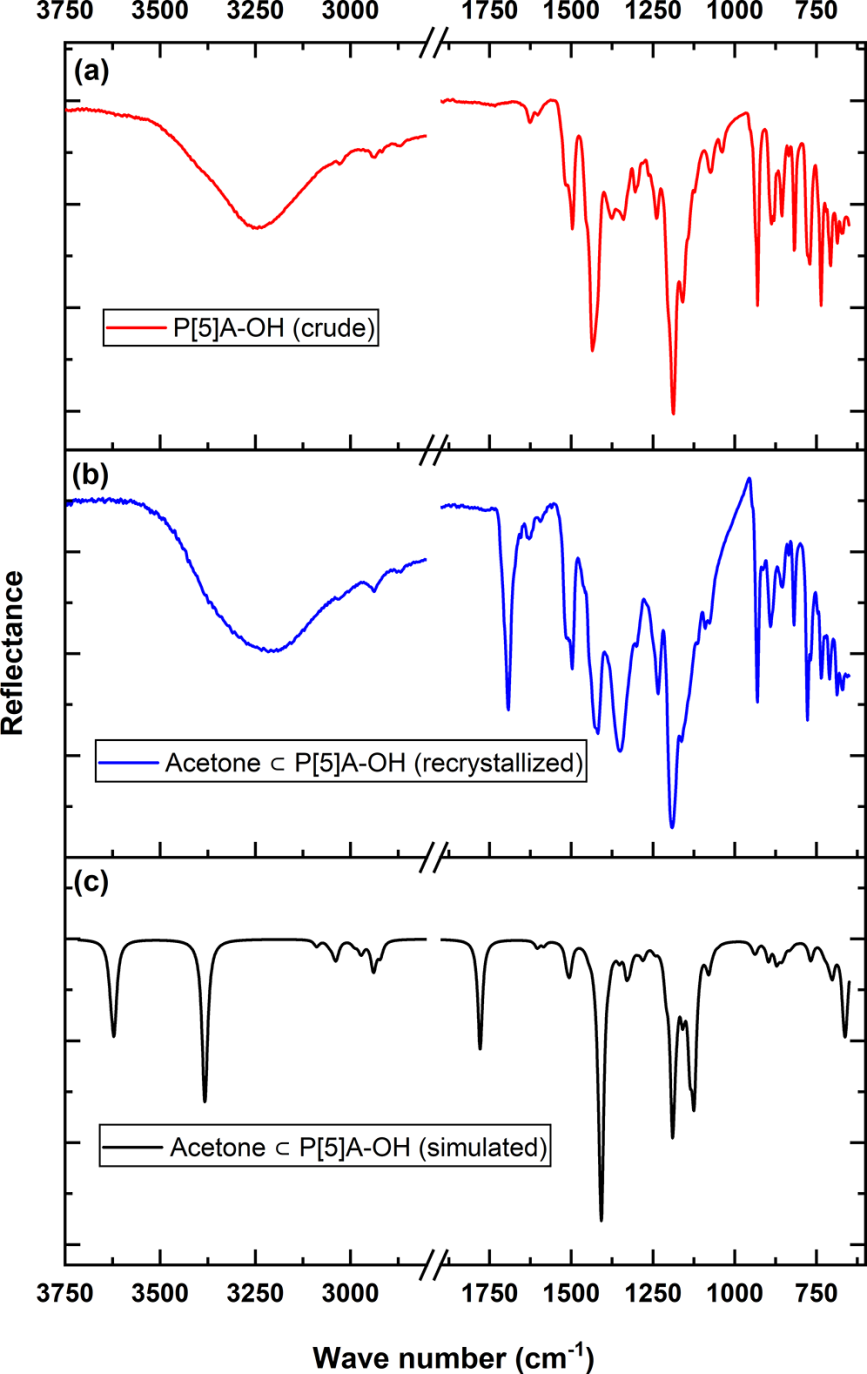
Comparison of IR spectra of (a) P[5]­A–OH (crude), (b) acetone ⊂ P[5]­A–OH (recrystallized), and (c) acetone ⊂ P[5]­A–OH (simulated).

The overestimation of the calculated O–H stretching frequencies is likely due to two factors: the harmonic approximation and the lack of explicit intermolecular hydrogen bonding in the computational model. First, the harmonic approximation often assumes that atomic vibrations behave like ideal harmonic oscillators; that means that the potential energy increases symmetrically as atoms move away from their equilibrium positions. However, real molecular vibrations, especially for bonds involving hydrogen like O–H, often exhibit anharmonic behavior, particularly at higher energies. This anharmonicity tends to lower the actual vibrational frequencies compared with what is predicted using the harmonic model, resulting in an overestimation in the calculated values. Second, our computational model simulates an isolated crystal structure without accounting for intermolecular hydrogen bonding. Under experimental conditions, P[5]­A–OH molecules participate in strong hydrogen bonds with neighboring molecules. These hydrogen bonds weaken the O–H bond, leading to a lower stretching frequency. Since this weakening effect is not included in the standard *ab initio* setup, the computed O–H stretching modes appear at higher (blue-shifted) frequencies than observed experimentally. Another important point is that the DFT simulation includes only a single unit with two acetone molecules absorbed in one P[5]­A–OH, which results in sharper IR peaks. In contrast, the experimental IR spectrum comes from a bulk supramolecular structure with many interacting groups. These interactions cause peak broadening and shifting, which can also lead to differences between the calculated and the experimental spectra.

In a previously reported study by Ogoshi et al.,^13^ the experimental investigations reveal the absorption of two acetone molecules within the cavity of P[5]­A–OH, that aligns mostly parallel to the macrocyclic axis. In our experimental study, we verified the absorption of acetone molecules by P[5]­A–OH. However, further investigation with density functional tight-binding (DFTB) computational analyses reveal a different arrangement of the absorbed acetone molecules, which are depicted in both top and side views, respectively (Figure 5a,5b). Specifically, the calculated results indicate that two acetone molecules assume an orientation nearly perpendicular to the P[5]­A–OH axis. This configuration is stabilized by two primary interactions: (1) Hydrogen bonding between the oxygen of acetone and the hydrogen of – OH groups of P[5]­A–OH and (2) CH−π interactions between the methyl hydrogen atoms of acetone and the electron-rich aromatic rings of the P[5]­A–OH. Together, these interactions orient the absorbed acetone molecules perpendicular to the axis of P[5]­A–OH. These results emphasize the complexity of host–guest interactions and the role of functional groups in supramolecular systems. Our findings align with Ogoshi’s report in confirming acetone absorption. They also provide new insights into the structural adaptability of P[5]­A–OH and the role of noncovalent forces in dictating guest orientation.

**Figure 5:**
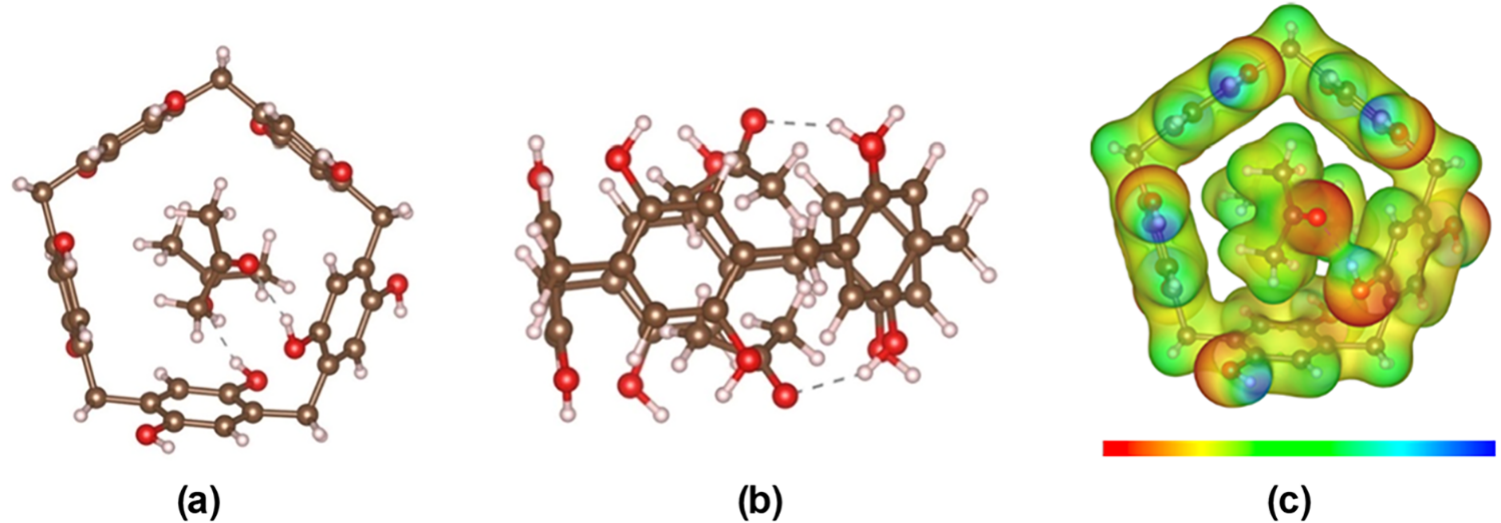
Absorption of two acetone molecules within the cavity of P[5]­A–OH: (a) top view and (b) side view (brown: C, red: O, pink: H), and (c) electrostatic potential map with isosurface level of 0.015, ranging from significant negative (red) to positive (blue).

A comparison of the calculated IR spectra of P[5]­A–OH before and after acetone absorption is illustrated in Figure 6. A new band at 1779 cm^–1^ emerged in the IR spectrum of P[5]­A–OH with absorbed acetone molecules (red lines), corresponding to the C=O stretching vibration. This band is absent in the IR spectrum of pristine P[5]­A–OH (green lines) and confirms the successful inclusion of acetone molecules in the host cavity. The computed C=O frequency shifting from 1727 cm^–1^ in free acetone (black lines) to 1779 cm^–1^ in the absorbed state, which is in agreement to the previous study of Alver et al.^51^ In free acetone, the C=O bond is mainly influenced by its intrinsic dipole moment and weak intermolecular interactions. Upon absorption into the cavity of P[5]­A–OH, the C=O bond also engages in hydrogen bonding with the hydroxyl (−OH) groups of the host structure. This interaction induces changes in the absorbed acetone molecules.

**Figure 6:**
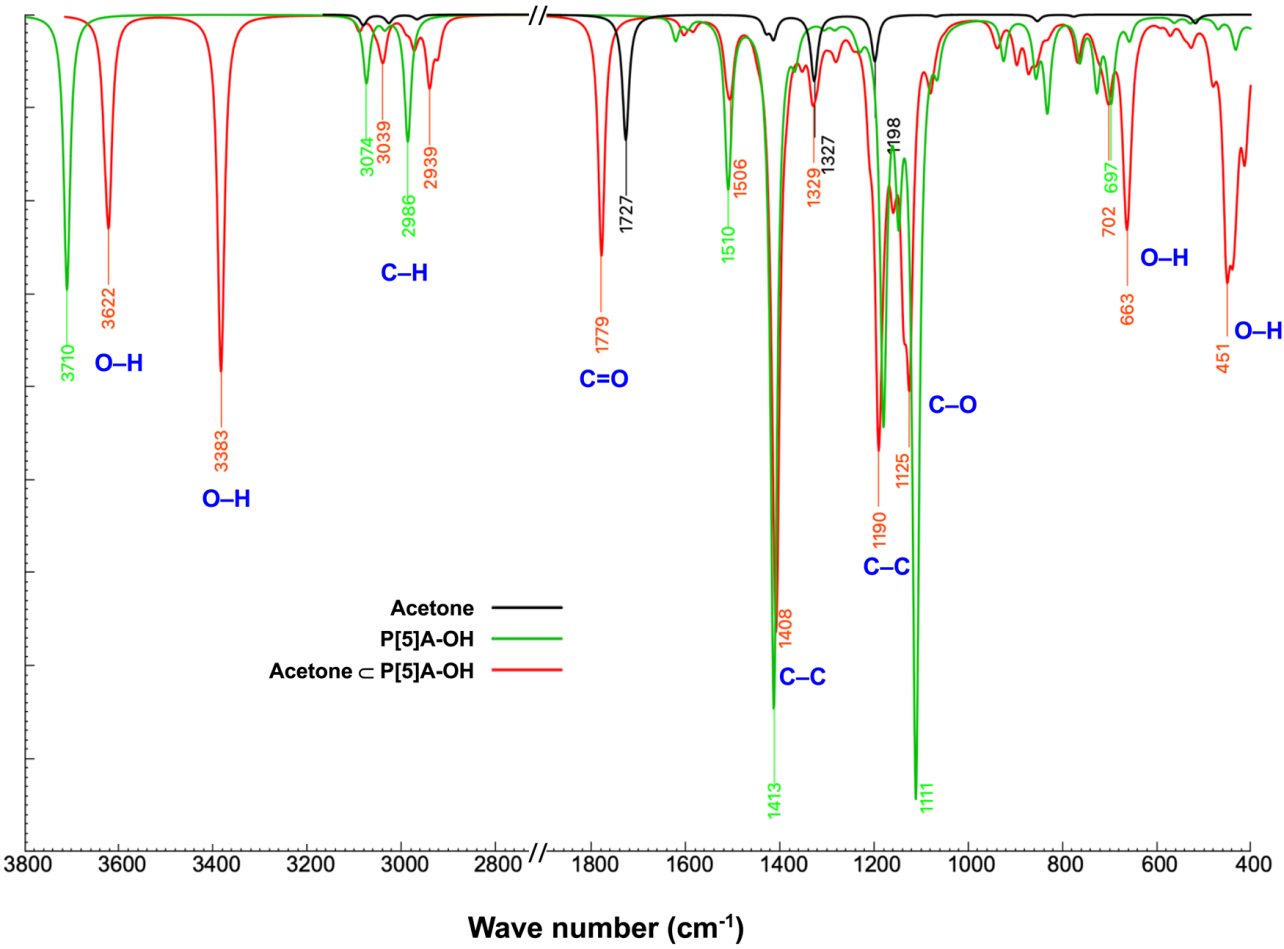
DFT simulation of the IR spectra of free acetone (black), P[5]­A–OH before acetone absorption (green), and P[5]­A–OH after acetone absorption (red).

Our computational results show a contraction in the C=O bond length from 1.24 Å in free acetone to 1.22 Å upon absorption, accompanied by a pronounced blue shift in the C=O stretching vibration of 52 cm^–1^ in the IR spectrum. The observed bond shortening and spectral shift are attributed to the charge redistribution within the C=O group induced by the host environment. As indicated by the electron density analysis (Table 2), the oxygen atom of the carbonyl group experiences an increase in electron density (7.06 → 7.13) upon absorption, while the carbon atom retains its original electron density (3.04 → 3.04). This redistribution enhances the polarization of the C=O bond, thereby reinforcing its double-bond character. The increased electron density on oxygen strengthens the bond by amplifying the electrostatic attraction between the carbon and oxygen nuclei, which ultimately results in bond contraction.

**Table 2:** Electron Density (in Atomic Units) of C and O Atoms in Free and Absorbed Acetone

atom	free acetone	absorbed acetone	electron redistribution
O	7.06	7.13	+0.07
C	3.04	3.04	0

The computational IR analysis of P[5]­A–OH before and after acetone absorption also reveals significant changes in the O–H stretching region. Before the absorption of acetone, a single sharp peak at 3710 cm^–1^ is observed (green lines), which shows the free or non-hydrogen-bonded −OH groups in P[5]­A–OH. This high wavenumber value is consistent with the stretching vibration of −OH groups in the absence of strong intermolecular interactions. After the absorption of acetone molecules, the original −OH peak shifts to a lower frequency at 3622 cm^–1^, and a new peak emerges at 3383 cm^–1^. This further confirms the hydrogen bonding interaction between C=O groups of acetone molecules, which act as hydrogen bond acceptors, and −OH groups of P[5]­A–OH serving as hydrogen bond donors. The distinct splitting into two peaks suggests the formation of two discrete hydrogen-bonding environments for −OH groups upon acetone inclusion. The red shift to 3383 cm^–1^ shows a strong hydrogen bond between certain −OH groups and the C=O group of acetone molecules. This could arise from direct linear hydrogen bonding, in which the −OH group is elongated and the O–H bond is weakened. The smaller shift to 3622 cm^–1^ corresponds to weaker hydrogen bonds, which are likely due to the interactions of other −OH groups with acetone molecules. This peak could represent secondary interactions where −OH groups experience partial electronic effects from nearby absorbed acetones without direct bonding.

The integration of experimental IR spectroscopy and scaled DFT calculations provides a framework for analyzing host–guest interactions in acetone ⊂ P[5]­A–OH systems. The scaling protocol bridges theoretical and experimental results, enabling precise assignment of vibrational modes and identification of absorption-induced electronic changes. The emergence of the C=O band at 1779 cm^–1^ serves as a spectroscopic identifier of acetone inclusion, while the shift and split of the −OH peak indicate the interaction between acetone and P[5]­A–OH occurs via the hydrogen bond between the −OH group of P[5]­A–OH and the C=O group of acetone. These findings highlight the role of functional groups in stabilizing guest molecules within pillararene hosts with broader implications for designing functional supramolecular materials.

### 3.3 Infrared Spectroscopy of P[5]­Q

To investigate the electronic and structural effects of TCE absorption on P[5]­Q, both experimental (red and blue) IR spectra and the DFT-simulated (black) IR spectra for the TCE ⊂ P[5]­Q complex were analyzed and compared (Figure 7). The C–H stretching region (∼3000 cm^–1^) exhibited two distinct peaks at 2994 and 3049 cm^–1^ in the experimental spectrum. In comparison, the DFT calculations predicted corresponding peaks at 2998 and 3004 cm^–1^. Notably, in the experimental spectra, vibrational modes in the 700–800 cm^–1^ region were assigned to C–Cl stretching vibrations of the guest molecule. In the simulation, the DFT predicted C–Cl stretching frequencies at lower values (500–600 cm^–1^). In contrast to the over estimation of the C–H peak at 3000 cm^–1^, C–Cl vibrations involve heavier atoms and occur at lower frequencies, where long-range and intermolecular effects become more significant. These are often underestimated in DFT,^52,53^ resulting in a red shift of the computed C–Cl stretching modes relative to the experimental observations.

**Figure 7:**
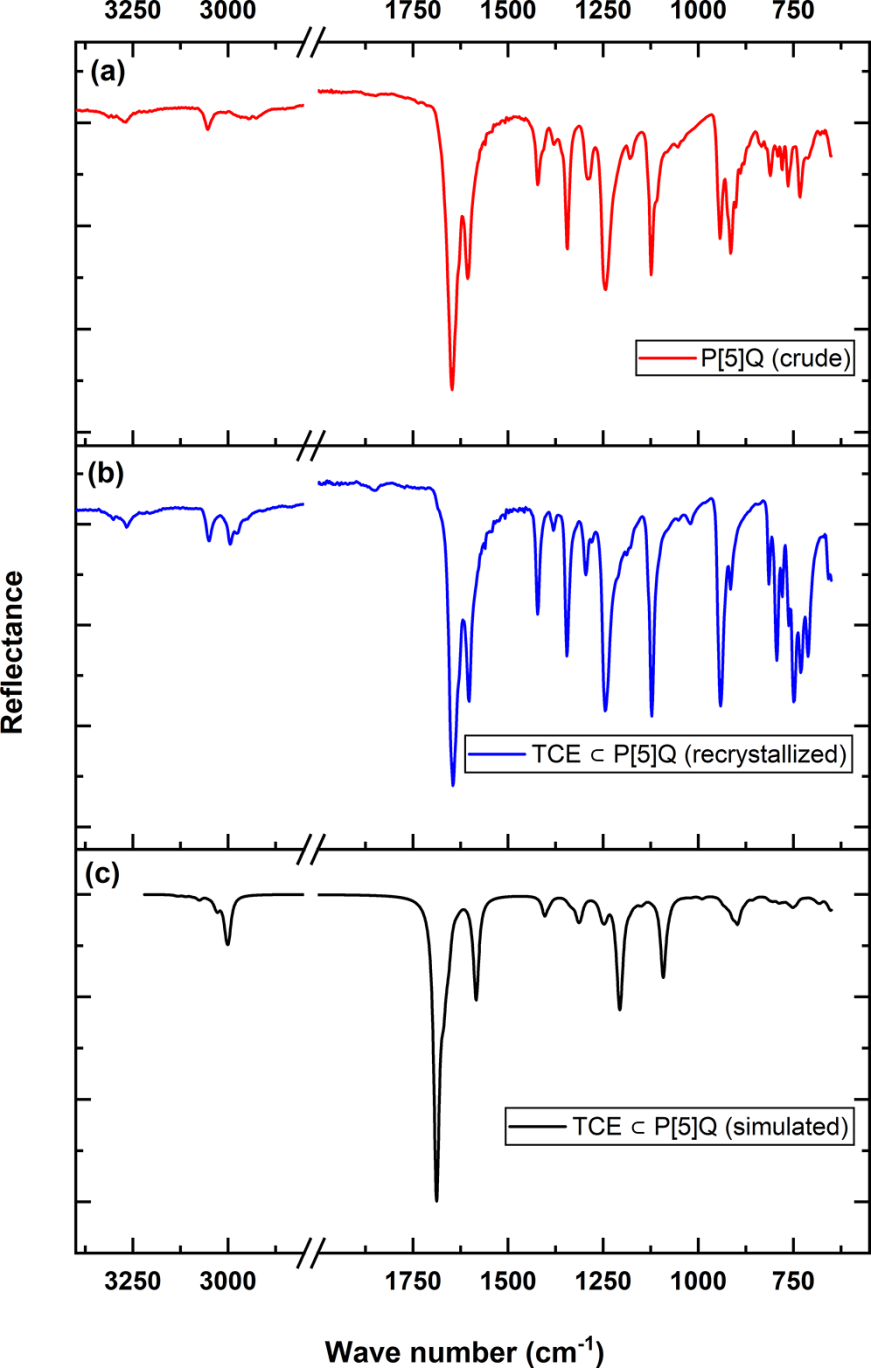
Comparison of IR spectra of (a) P[5]­Q (crude), (b) TCE ⊂ P[5]­Q (recrystallized), and (c) TCE ⊂ P[5]­Q (simulated).

The absorption of TCE molecules within the macrocyclic host cavity of P[5]­Q was modeled by DFTB+ simulation and is depicted in both top and side views (Figure 8a,8b). DFTB calculations revealed that the absorption is stabilized by hydrogen bonds between hydrogen atoms of TCE and oxygen atoms of P[5]­Q. The calculated binding energy of 1.1 eV per two TCE molecules (0.55 eV/molecule) is consistent with moderately strong hydrogen bond-driven host–guest systems and aligns with previously reported values for analogous supramolecular complexes.^54,55^

**Figure 8:**
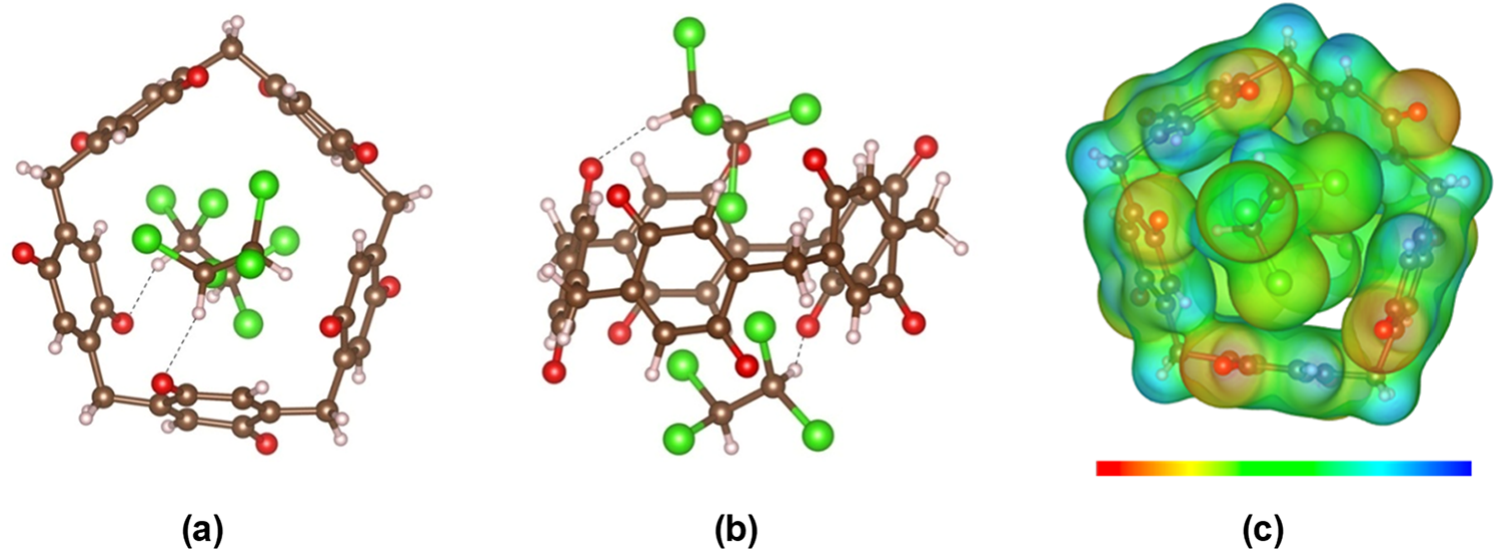
Absorption of two TCE molecules at the external cavity of P[5]­Q: (a) top view and (b) side view (brown: C, red: O, pink: H and green: Cl), and (c) electrostatic potential map with isosurface level of 0.01, ranging from significant negative (red) to positive (blue).

Figures 5c and 8c show the electrostatic potential maps (MEP-display by VESTA^56^) of two acetone molecules and two TCE molecules absorbed by P[5]­A–OH and P[5]­Q, respectively. From Figure 5c, it can be seen that the main interaction between acetone and P[5]­A–OH is a hydrogen bond between the oxygen atom of acetone and hydrogen of the −OH group of P[5]­A–OH. In addition, the electron-poor C–H region of acetone (greenish in MEP) also interacts with the π-electron-rich benzene ring (yellow and red). As a result, both acetone molecules are located inside the cavity of P[5]­A–OH. In contrast, Figure 8c of the TCE ⊂ P[5]­Q system shows that the main interactions occur between two C–H groups (blue) of TCE and oxygen atoms of P[5]­Q (red), as well as between the chlorine atoms of TCE and oxygen atoms of P[5]­Q. These interactions take place outside the P[5]­Q cavity, suggesting that TCE is absorbed externally.

The calculated IR spectra of P[5]­Q before and after TCE absorption (Figure 9) provide a comparative basis for evaluating the changes in the characteristic peaks attributable to guest incorporation. In the simulated spectrum of host–guest complex, a key observation is the emergence of new C–H stretching peaks near 3000 cm^–1^, which were absent in the spectra of both pure P[5]­Q and TCE. These newly formed peaks are due to the hydrogen bond between C=O of P[5]­Q and C–H of TCE. The strong intensities in the simulated spectrum of host–guest complex further validate the interaction between P[5]­Q and TCE. Additionally, the calculated IR spectra show slight shifts in the mid-frequency region upon TCE absorption. Specifically, the C=O peaks of P[5]­Q at 1716 cm^–1^ shifted to 1688 cm^–1^ due to the weak hydrogen bond between TCE and P[5]­Q, while the C–C peaks at 1231 and 1114 cm^–1^ are red-shifted to 1206 and 1092 cm^–1^.

**Figure 9:**
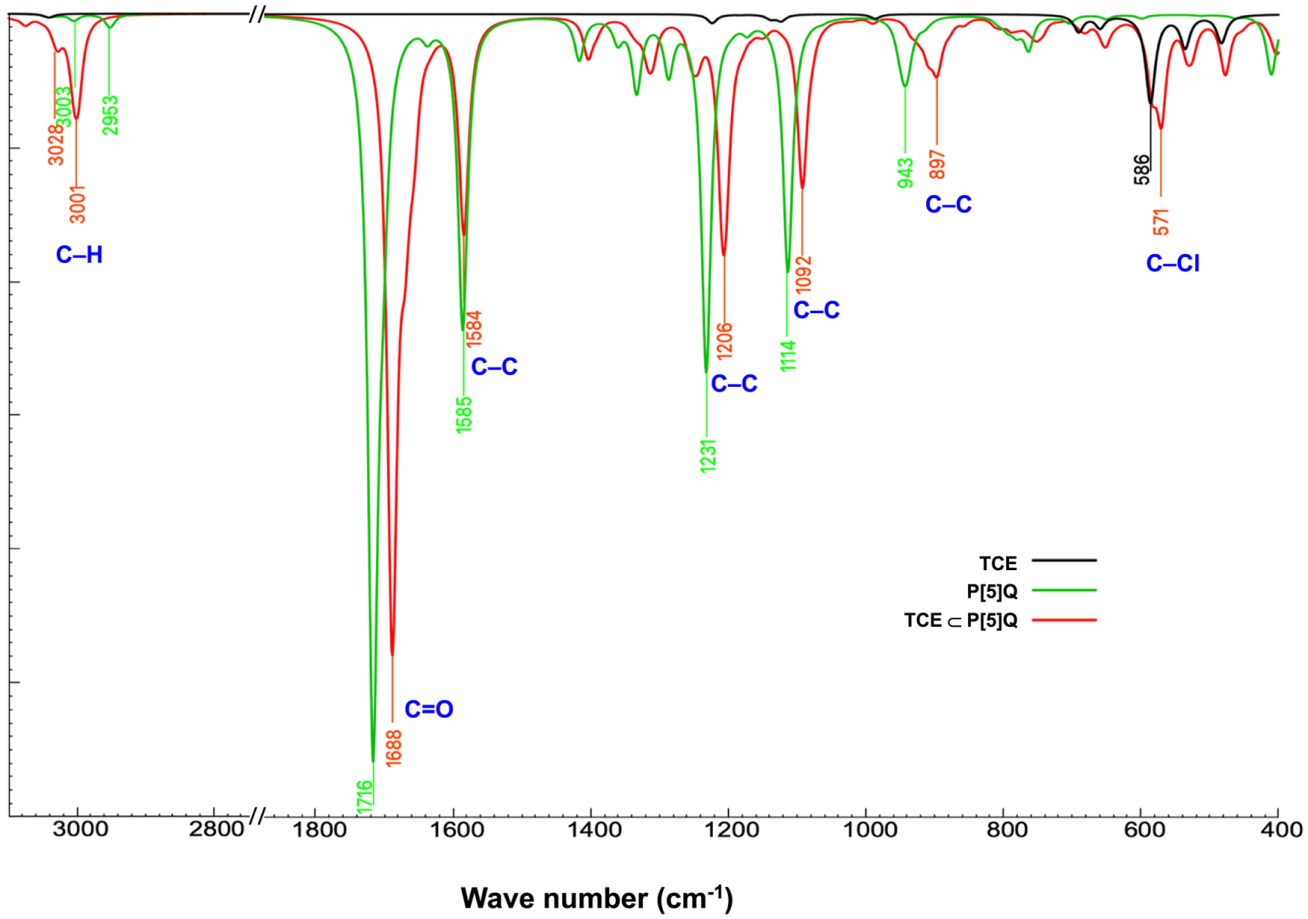
DFT simulation of the IR spectrum of free TCE (black), P[5]­Q before TCE absorption (green), and P[5]­Q after TCE absorption (red).

The integration of experimental IR spectroscopy with scaled DFT calculations provides a framework for analyzing the host–guest interactions in TCE ⊂ P[5]­Q systems. The observed shift of the C–Cl band to lower frequencies serves as a spectroscopic identifier of the guest complexation, while the presence of new C–H stretching peaks near 3000 cm^–1^ confirms the absorption via hydrogen bonding. These findings underscore the critical role of hydrogen bonding in stabilizing the guest molecules within pillararene hosts and offer valuable insights for the design of functional supramolecular materials with tailored absorption properties.

## 4 Conclusions

The experimentally observed host–guest complexation of P[5]­A–OH with acetone and P[5]­Q with TCE in a 1:2 stoichiometric ratio was further validated by computational modeling. Notably, the computational results revealed that P[5]­A–OH forms an inclusion complex with two acetone molecules located in the central cavity, while P[5]­Q forms a complex with two TCE molecules binding external to the cavity. The solid complexes are stable under a high vacuum at room temperature. However, acetone and TCE undergo exchange in the polar solvents needed for NMR analysis. We have carried out the first comprehensive computational mechanistic studies on the complexation behavior of solvent molecules into these cage compounds. In P[5]­A–OH, acetone molecules were absorbed and stabilized inside the cavity by hydrogen bonding and CH−π interactions, inducing a C=O blue shift and bond contraction due to electron density redistribution. The observed splitting of the O–H stretching peak confirms the presence of heterogeneous hydrogen bonding environments. For P[5]­Q, TCE absorption relies on hydrogen bonds, generating the shift in IR peaks and C–Cl/C=O vibrations. The findings in this study demonstrate pillararenes’ structural adaptability and the role of functional groups in guest stabilization. The synergy of experimental and scaled computational methods offers a strong framework for analyzing host–guest systems that helps to advance the design of functional supramolecular materials for gas absorption applications.

## Supplementary Material



## Notes

Detailed experimental procedures, ^1^H and ^13^C NMR spectra of the synthesized compounds, and density of states diagrams (PDF)
