# Supplementary material for: Synthesis and Characterization of Solvent-Complexes of per-Hydroxy Pillar[5]arene and Pillar[5]quinone: Experimental and Computational Insights
Source: ACS Omega. 2026 Mar 4;11(10):16088–97. doi: 10.1021/acsomega.5c10756 (PMC13000612; doi:10.1021/acsomega.5c10756)
Supplement: Supplementary file 1 [file ao5c10756_si_001.pdf]

Supporting Information

Synthesis and characterization of solvent-complexes of *per*-hydroxy pillar[5]arene and pillar[5]quinone: Experimental and computational insights

Venkatesh Bollabathini,*,[1] Quoc D. Ho,[2] Eva Rauls,[2] Kåre B. Jørgensen[1]

[1] Department of Chemistry, Bioscience and Environmental Engineering, Faculty of Science and Technology, University of Stavanger, P.O Box 8600 Forus, N-4036 Stavanger, Norway

[2] Department of Mathematics and Physics, Faculty of Science and Technology, University of Stavanger, P.O Box 8600 Forus, N-4036 Stavanger, Norway

*Corresponding Author: Venkatesh Bollabathini, Email: venkatesh.bollabathini@uis.no

Table of Contents

1. General information S2
2. Synthesis of compounds S2
   2.1. Synthesis of 1,4-dimethoxypillar[5]arene S2
   2.2. Synthesis of *per*-hydroxy pillar[5]arene S3
   2.3. Synthesis of pillar[5]quinone S4
3. ^{1}H and ^{13}C NMR spectra S6
4. Density of states diagrams S11
5. References S12

1. General information

All chemicals were purchased from commercial sources (Merck, VWR, TCI and Thermo Fischer Scientific) and used without further purification. The solvents were either employed as purchased or dried over molecular sieves (4 Å). The reactions were performed in oven dried glassware under nitrogen atmosphere or in open air according to the synthetic procedures. Thin layer chromatography (TLC) was performed on silica gel 60 F_{254} aluminium sheets, purchased from Merck and visualized by UV light. The ^{1}H and ^{13}C NMR were recorded with 400 MHz Bruker AVANCE III spectrometer at 400 MHz and 100 MHz, respectively. The chemical shifts were expressed in parts per million (ppm) and the residual undeuterated solvent peaks were used as internal references. The ^{1}H and ^{13}C NMR chemical shifts were calibrated relative to the residual solvent peaks of $CDCl_3$ (δ 7.26 and 77.16), DMSO-*d6* (δ 2.50 and 39.5), and TFA-*d* (δ 11.50 and 164.2), respectively. Melting points were measured on the Büchi MP-3 melting point apparatus. Infrared (IR) spectra were measured as neat compounds on an Agilent Carey 630 FTIR spectrophotometer equipped with an attenuated total reflectance (ATR) sampling module. HRMS were measured on JMS T100 GCAccuTOFTM EI-TOF from JEOL.

2. Synthesis of compounds

2.1. 1,4-Dimethoxypillar[5]arene (DMP[5]A)

The synthesis was carried out in accordance with a previously reported procedure.[S1] An oven dried round bottom flask was charged with 1,4-dimethoxybenzene (2.764 g, 20.0 mmol, 1.0 equiv.) and paraformaldehyde (1.802 g, 60.0 mmol, 3.0 equiv.) followed by nitrogen gas backfilling for 10 min. To this, anhydrous 1,2-dichloroethane (DCE) (40 mL) was added and the solution was stirred for 10 min at room temperature. Subsequently, boron trifluoride diethyl etherate ($BF_3{\cdot}OEt_2$) (2.47 mL, 20.0 mmol, 1.0 equiv.) was added dropwise. The reaction mixture turned into a green colour within few minutes and continued to stir for 0.5 h at 30 °C. After completion of the reaction (monitored by TLC), the solution was poured into methanol (200 mL) at 0 °C and the resulting precipitate was collected by vacuum filtration and dried under high vacuum for overnight. For further purification, the crude product was subjected to recrystallization by dissolving it in chloroform (60 mL) in an Erlenmeyer flask followed by the addition of an equal amount of acetone (60 mL). Afterwards, the solution was placed in a freezer at –20 °C for 24 h and the resulting precipitate was collected by vacuum filtration. To precipitate

out the complete product, the filtrate was concentrated under reduced pressure and the residue subjected to second recrystallization in a minimum solvent mixture of chloroform and acetone. The combined precipitates were dried under high vacuum for overnight to yield DMP[5]A as a white solid (2.843 g, 94%).

^{1}H NMR (400 MHz, $CDCl_3$): δ 6.84 (s, Ar-**H**, 10H), 3.76 (s, −C**H$_2$** bridge, 10H), 3.71 (s, −OC**H$_3$**, 30H). ^{13}C NMR (100 MHz, $CDCl_3$): δ 150.6 (−**C**−OCH_3, Ar), 128.3 (−**C**−CH_2, Ar), 113.6 (−**C**H, Ar), 55.6 (−O**C**H_3), 29.4 (−**C**H_2 bridge). HRMS (ESI) *m/z*: $[M+H]^+$ Calcd for $C_{45}H_{51}O_{10}$ 751.3477; Found 751.3476. mp: 248.2–249. 6 °C. IR (ATR, cm^{-1}): 2985, 2933, 1495, 1396, 1206, 1044. The spectral characterization data are in accordance with the literature.[S1]

2.2. *per*-Hydroxy pillar[5]arene (P[5]A-OH)

An oven dried round bottom flask was charged with 1,4-dimethoxypillar[5]arene (2.0 g, 2.66 mmol) and backfilled with nitrogen gas for 10 min. To this, anhydrous dichloromethane (150 mL) was added and the solution was stirred for 10 min. While stirring, boron tribromide (BBr_3) (3.8 mL, 40 mmol) was added dropwise to the reaction flask at –78 °C, which was gradually allowed to reach room temperature and continued to stir for 72 h. After completion of the reaction (monitored by TLC), it was quenched with ice cold water (100 mL) and the resulting precipitate was stirred for 1 h at room temperature. The precipitate was collected by vacuum filtration and washed with 0.5 M HCl (2 × 100 mL) followed by chloroform (2 × 100 mL). The resulting solid was left to dry under high vacuum for overnight to yield a white solid (1.518 g).

For further purification, the crude product was subjected to a recrystallization process. Initially, the compound was taken in an Erlenmeyer flask (100 mL) and 2-3 boiling chips were added to it. To this, hot acetone (approx. 40 mL) was added in small portions and the mixture was heated to boiling on a hot plate to ensure complete dissolution of the solid. On the other hand, an Erlenmeyer flask (100 mL) containing a small portion of fresh hot acetone (approx. 10 mL) was taken (where the filtrate from hot filtration to be collected) and 2-3 boiling chips were added to it. For hot filtration, a short stem funnel with fluted filter paper was placed on top of the flask. During this process, the whole set up was placed on a heating plate, allowing the funnel to be heated by the rising solvent vapours. The hot filtration was carried out very carefully by preheating the fluted filter paper in the funnel with few drops of hot acetone to avoid any crystallization of solid in the filter paper. After the solution was hot filtered, the filtrate was left to cool at room temperature for 1 h, purged with nitrogen and placed in

refrigerator at +4 °C until the complete product was precipitated out. The obtained precipitate was collected by vacuum filtration and further dried under high vacuum overnight to yield a white crystalline solid (1.485 g, 91% (Taking into account the absorbed acetone molecules)).

^{1}H NMR (400 MHz, DMSO-*d6*): δ 8.44 (s, −O**H**, 10H), 6.57 (s, Ar-**H**, 10H), 3.43 (s, −C**H**$_2$ bridge, 10H). ^{13}C NMR (100 MHz, DMSO-*d6*): δ 146.1 (−**C**−OH, Ar), 126.4 (−**C**−CH$_2$, Ar), 117. 3 (−**C**H, Ar), 29.1 (−**C**H$_2$ bridge). HRMS (ESI) *m/z*: $[M+Na]^+$ Calcd for $C_{35}H_{30}O_{10}Na$ 633.1731; Found 633.1732. mp: The compound decomposed after 246 °C. IR (ATR, cm^{-1}): 3216 (br, −OH), 2938, 1692, 1497, 1418, 1191. The spectral characterization data are in accordance with the literature.[S1]

2.3. Pillar[5]quinone (P[5]Q)

The synthesis of P[5]Q was carried out according to our previously reported procedure.[S2] A round bottom flask was charged with 1,4-dimethoxypillar[5]arene (500 mg, 0.66 mmol) in MeCN (12 mL). The solution was stirred for 10 min at room temperature. A solution of CAN (5.48 g, 9.98 mmol) in H_2O (12 mL) was added drop wise and the reaction mixture was stirred for 0.5 h at room temperature. After the completion of the reaction (monitored by TLC), MeCN was removed under reduced pressure. The resulting precipitate was washed with distilled H_2O (3 × 30 mL), filtered and subsequently washed with MeOH (2 × 20 mL) to obtain the product P[5]Q as a yellowish solid (368 mg).

The synthesized product was further purified by recrystallization. Initially, the compound was introduced into an Erlenmeyer flask (50 mL) containing 2-3 boiling chips. Subsequently, hot 1,1,2,2-tetrachloroethane (TCE) (approx. 10 mL) was added and the resulting mixture was heated to boil until the solid was completely dissolved. A fresh hot TCE (approx. 2 mL) was taken in another Erlenmeyer flask (50 mL) (where the filtrate from hot filtration to be collected) containing 2-3 boiling chips. For hot filtration, a short stem funnel with fluted filter paper was placed on top of the flask. Prior to hot filtration, it was preheated with hot TCE to avoid any crystallization in the filter paper. During this process, the whole set up was placed on a heating plate, allowing the funnel to be heated by the rising solvent vapours. After the careful hot filtration of solution, the reddish coloured clear filtrate was left to cool at room temperature for 1 h then placed in a refrigerator at +4 °C overnight until the complete product was precipitated out. The obtained precipitate was collected by filtration, washed with cold MeOH and dried

under high vacuum overnight to yield a light-weight yellowish solid (351 mg, 88% (Taking into account the absorbed TCE molecules)).

^{1}H NMR (400 MHz, TFA-*d*): δ 7.02 (s, −C**H**, Q, 10H), 3.70 (s, −C**H₂** bridge, 10 H). ^{13}C NMR (100 MHz, TFA-*d*): δ 190.5 (−**C**=O, Q), 146.8 (−**C**−CH_2, Q), 138.1 (−**C**H, Q), 28.0 (−**C**H_2 bridge). HRMS (ESI) *m/z*: $[M]^-$ Calcd for $C_{35}H_{20}O_{10}$ 600.1062; Found 600.1059. mp: The compound decomposes above 250 °C. IR (ATR, cm^{-1}): 1645, 1603, 1421, 1345, 1295, 1244, 1122, 941, 915. The spectral characterization data are in accordance with the literature.[S3]

3. ^{1}H and ^{13}C NMR spectra

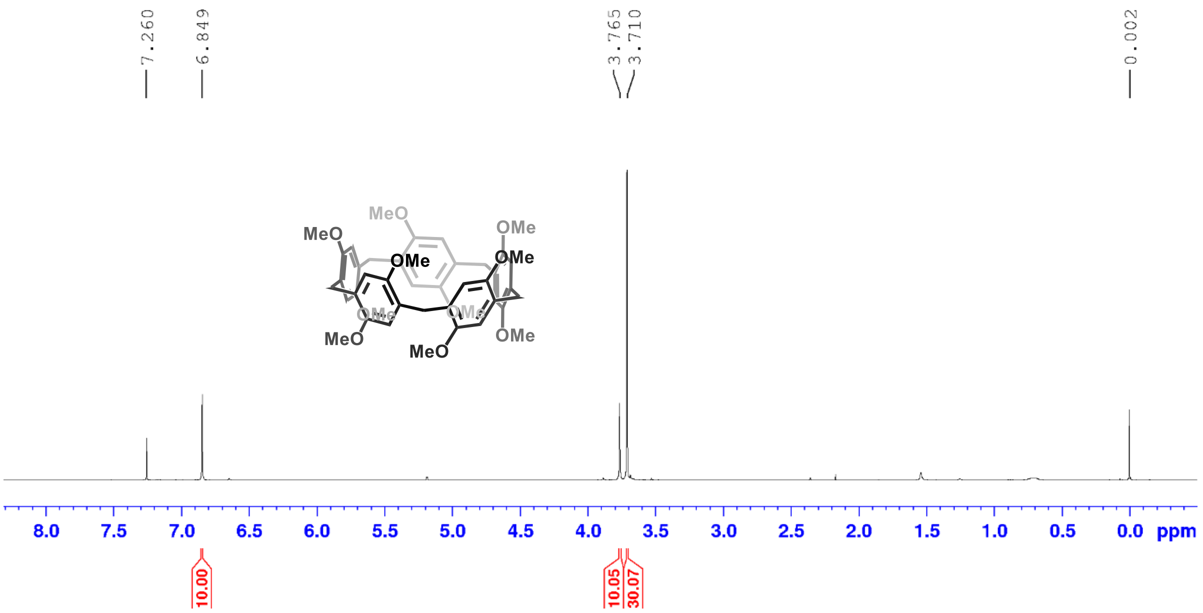

Figure S1. ^{1}H NMR of DMP[5]A (400 MHz, $CDCl_3$)

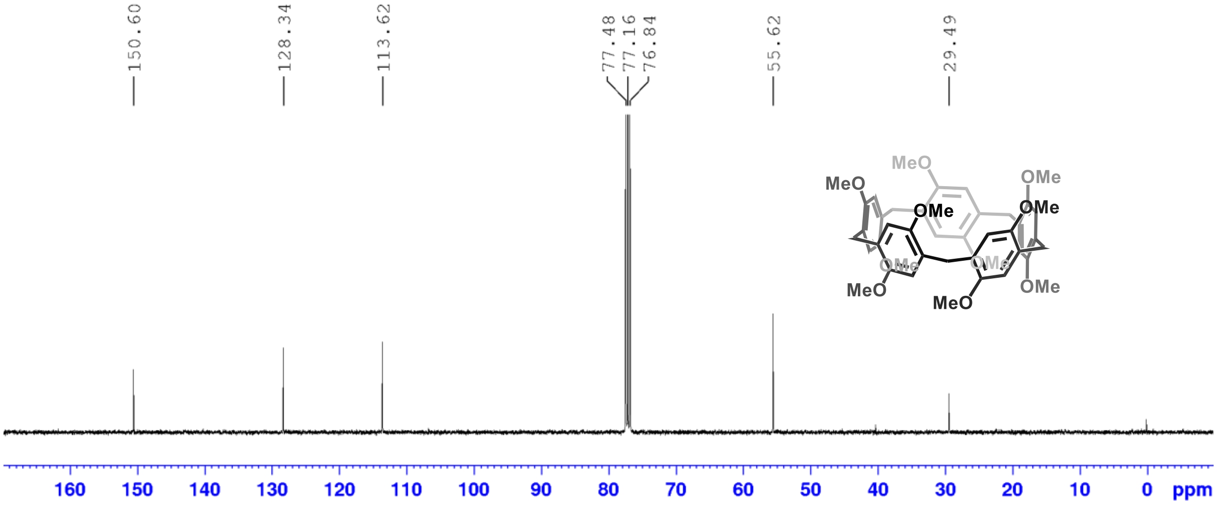

Figure S2. ^{13}C NMR of DMP[5]A (100 MHz, $CDCl_3$)

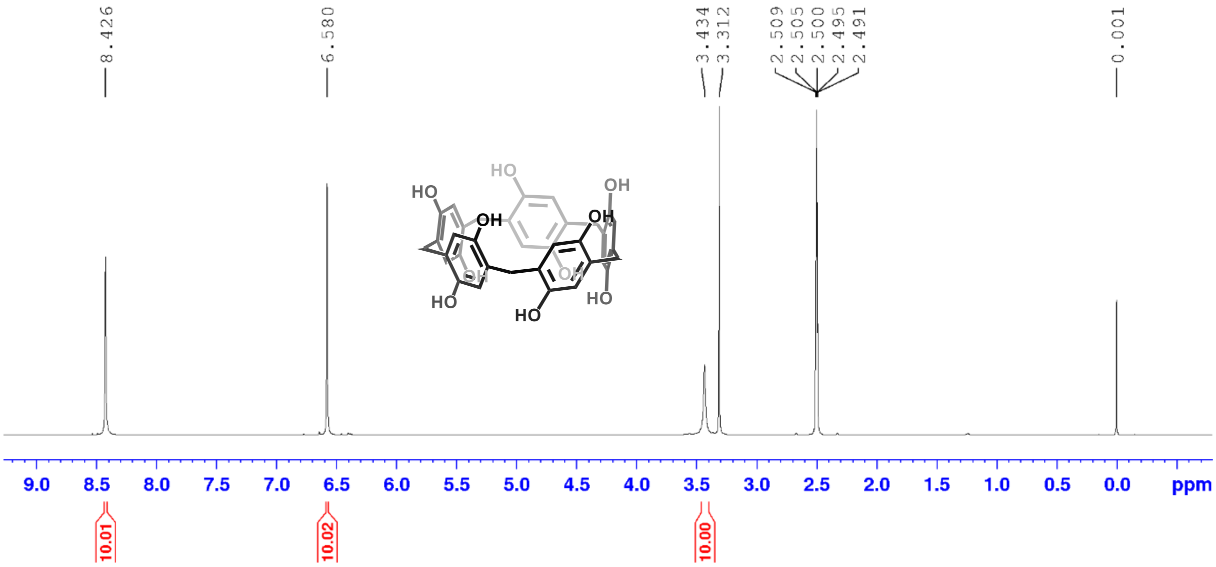

Figure S3. ^{1}H NMR of crude P[5]A-OH (400 MHz, DMSO-*d6*)

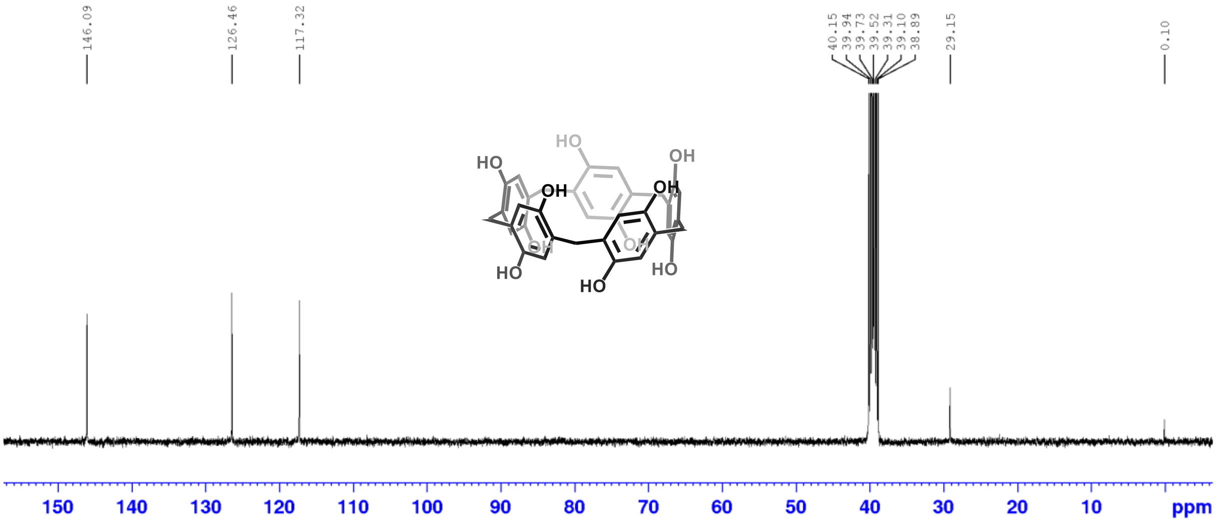

Figure S4. ^{13}C NMR of crude P[5]A-OH (100 MHz, DMSO-*d6*)

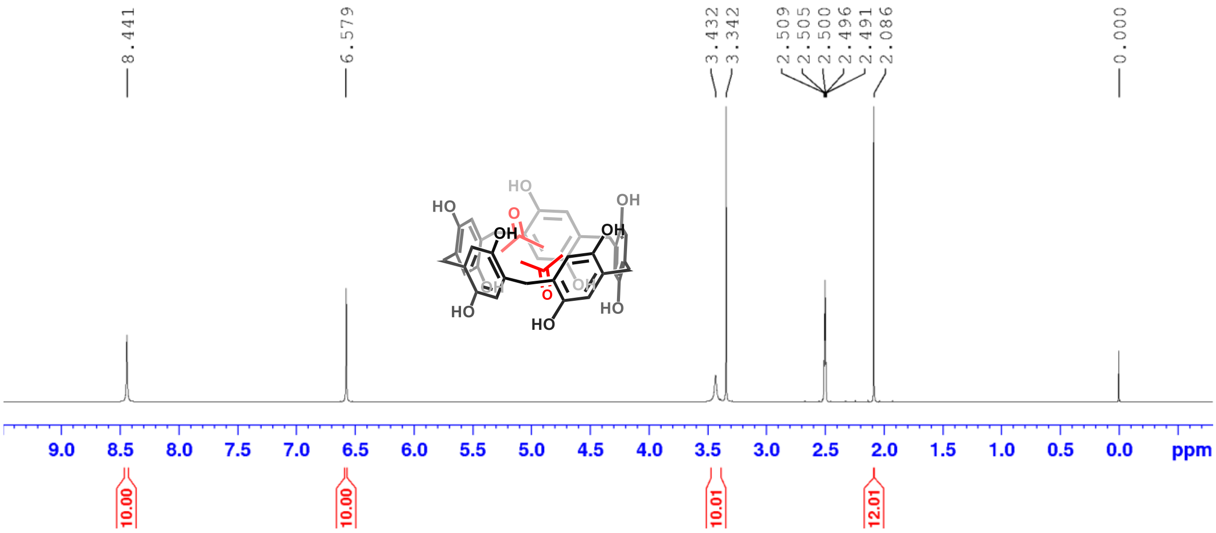

Figure S5. ^{1}H NMR of recrystallized P[5]A-OH (400 MHz, DMSO-*d6*)

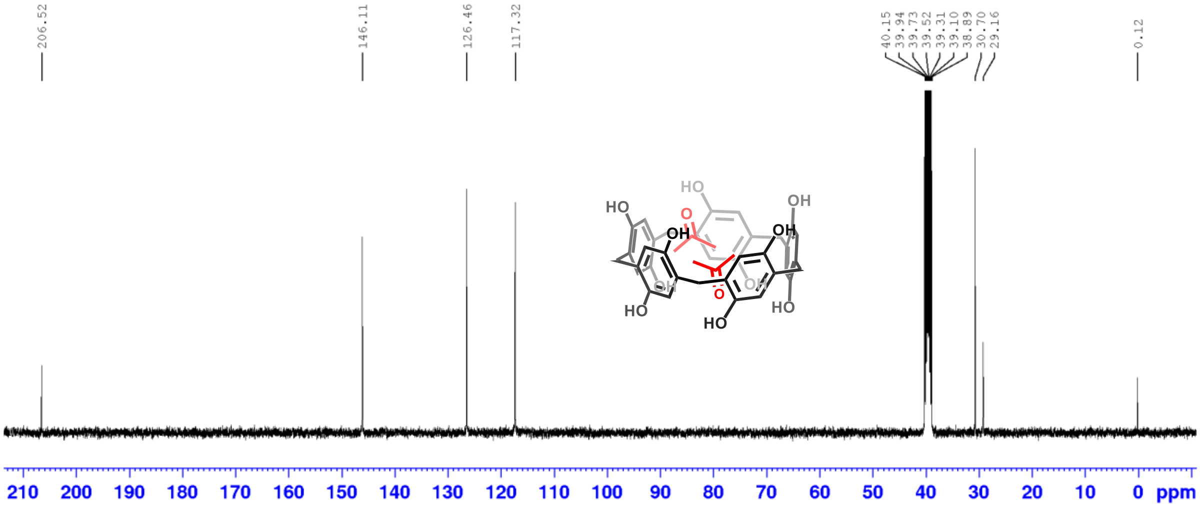

Figure S6. ^{13}C NMR of recrystallized P[5]A-OH (100 MHz, DMSO-*d6*)

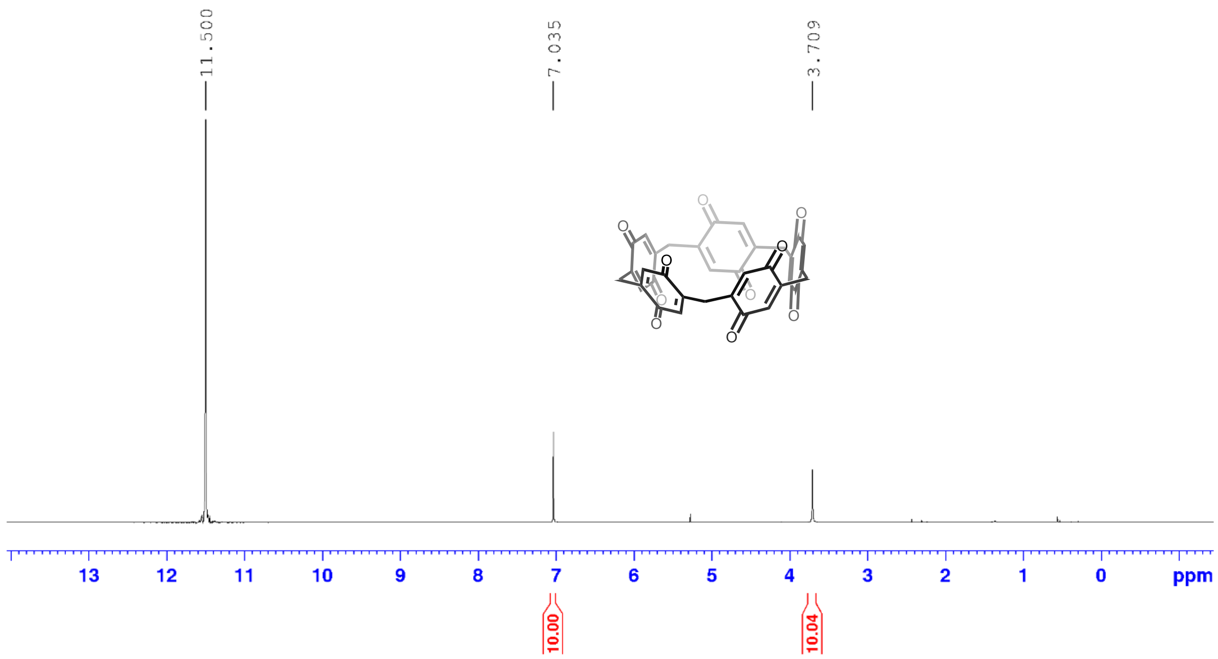

Figure S7. ^{1}H NMR of crude P[5]Q (400 MHz, TFA-*d*)

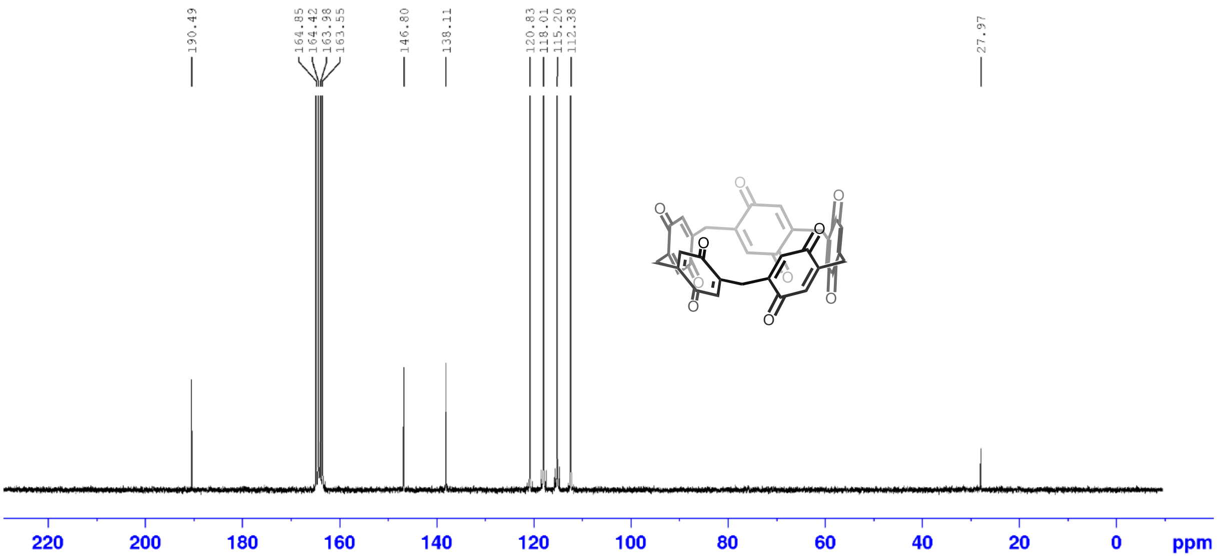

Figure S8. ^{13}C NMR of crude P[5]Q (100 MHz, TFA-*d*)

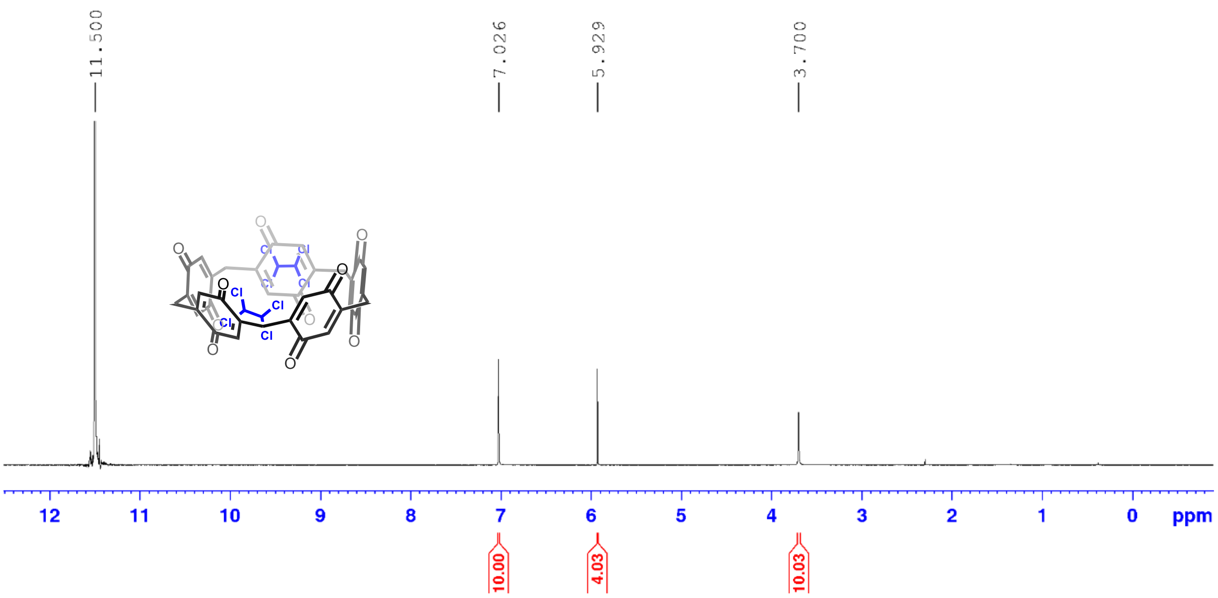

Figure S9. ^{1}H NMR of recrystallized P[5]Q (400 MHz, TFA-*d*)

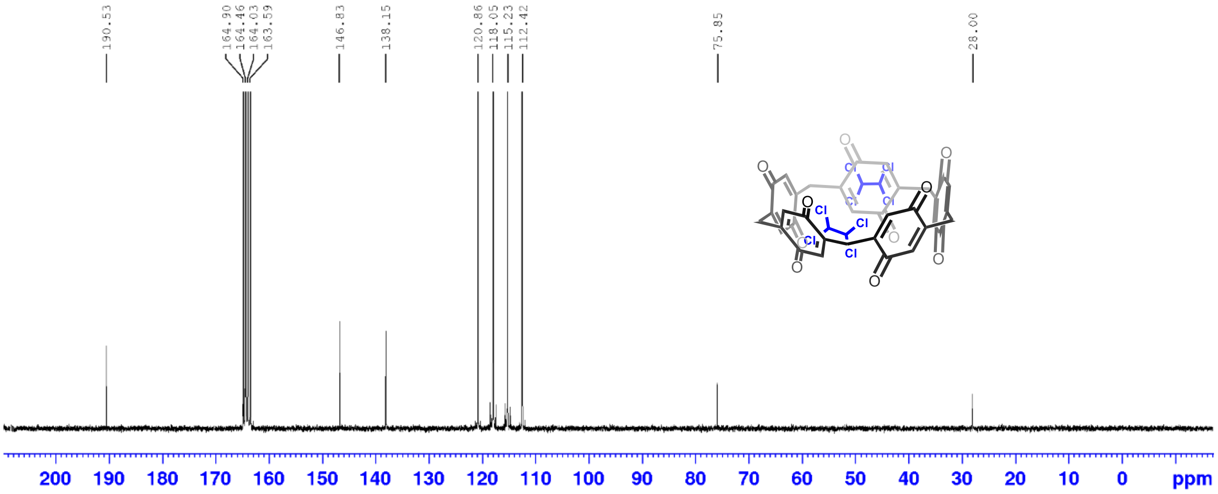

Figure S10. ^{13}C NMR of recrystallized P[5]Q (100 MHz, TFA-*d*)

4. Density of states diagrams

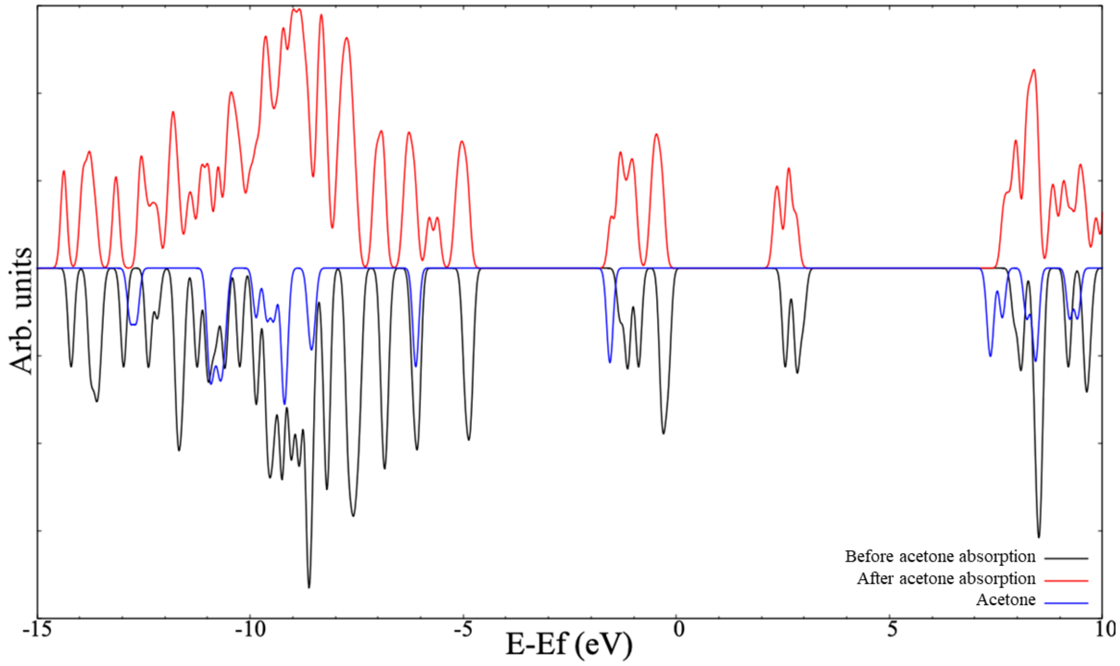

Figure S11. Density of states before and after absorption of acetone by P[5]A-OH

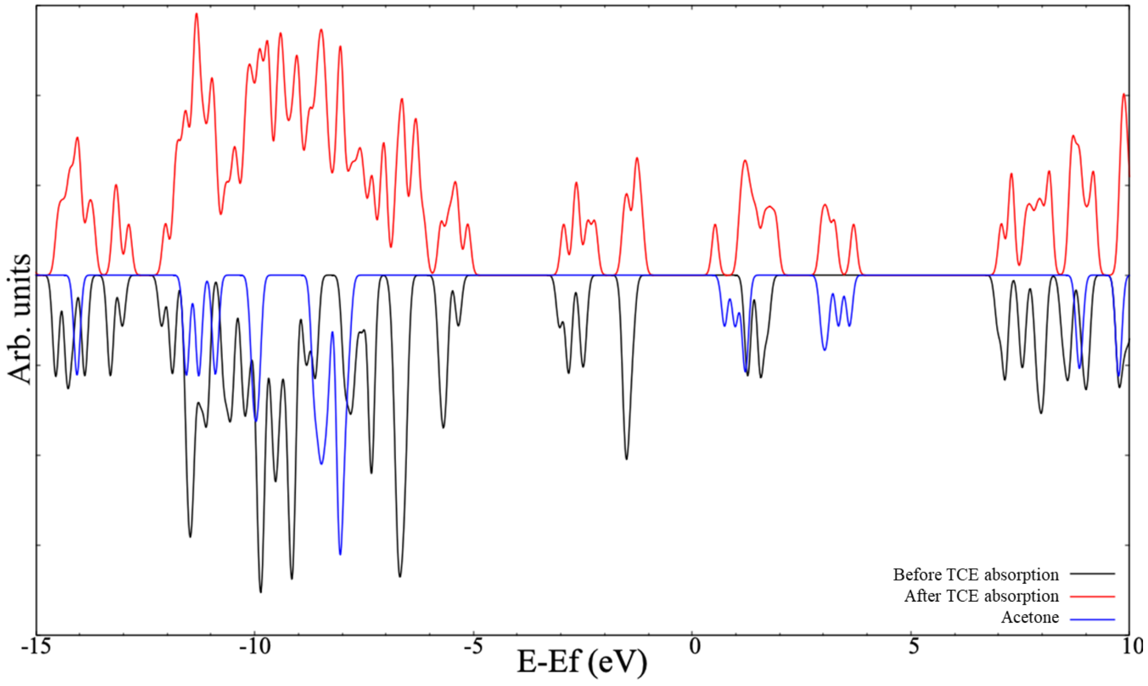

Figure S12. Density of states before and after absorption of TCE by P[5]Q

5. References

(S1) Ogoshi, T.; Aoki, T.; Kitajima, K.; Fujinami, S.; Yamagishi, T.; Nakamoto, Y. Facile, Rapid, and High-Yield Synthesis of Pillar[5]Arene from Commercially Available Reagents and Its X-Ray Crystal Structure. *J. Org. Chem.* **2011**, *76* (1), 328–331. https://doi.org/10.1021/jo1020823.

(S2) Bollabathini, V.; Jørgensen, K. B. Direct Synthesis of Pillar[5]Quinone and Pillar[6]Quinone by CAN Oxidation of Alkylated Pillararenes. *Tetrahedron Lett.* **2025**, *169*, 155739. https://doi.org/10.1016/j.tetlet.2025.155739.

(S3) Shivakumar, K. I.; Sanjayan, G. J. An Easy and Multigram-Scale Synthesis of Pillar[5]Quinone by the Hypervalent Iodine Oxidation of 1,4-Dimethoxypillar[5]Arene. *Synth.* **2013**, *45* (07), 896–898. https://doi.org/10.1055/s-0032-1318390.
